# An Account of the North Kensington Friendly Workers among the Poor

**Published:** 1892-09-24

**Authors:** C. Furley-Smith


### THE CURE OF POVERTY,

### AN ACCOUNT OF THE NORTH KENSINGTON FRIENDLY WORKERS AMONG THE POOR

—(*continued from page* 416).

By Mrs. C. FURLEY SMITH.

These are only a few cases selected from the records of the Association, and selected as being specially illustrative of the points on which it differs from other charitable societies. One of these is, how much more was given in thought, work, and personal attention than in money? This was all along a special feature of the Friendly Workers. Three of the rules ran as follow:—

6. No relief in money will be given unless it be shown to the satisfaction of the Secretary that employment cannot be obtained.

7. Relief in money will only be given in the following cases :—

(*a*). When the distress is shown to be exceptional.

(*b*). When it is required to keep a man on his benefit club, or reinstate him where his membership has recently run out.

(*c*). When it his required to redeem necessary articles that have been pawned, or to provide tools.

8. When relief in money is granted the Friendly Worker, on whose recommendation the grant was made, or the visitor, or member of the Committee who acts as almoner, will be required to see, as far as may be possible, that it is properly applied, and that the case is visited again within a week from payment.

The strictest watch was thus kept over the money entrusted to the Friendly Workers, and though grants were obtained in several cases from other charitable funds, the principle of helping the poor to help themselves was steadily adhered to. The result is that in September, 1891, when the Association had been in existence for two years and three months, 150 families, numbering in all about 700 souls, had been helped in one way or another—and most of them in a substantial and permanent way—at a total cost of £235 8s.

The balance-sheet is subjoined:

Statement of Receipts and Expenditure from July, 1889, to September, 1891 (Two Years and Three Months).

| Dr. | £ | s. | d. |
|---|---|---|---|
| To Received by Subscriptions and Donations ... ... ... | 247 | 0 | 6 |
| " Grants made on our recommendation, by Societies, &c., to meet Special Cases ... ... ... ... | 32 | 15 | 1 |
| | £279 | 15 | 7 |

| Cr. | £ | s. | d. | £ | s. | d. |
|---|---|---|---|---|---|---|
| By Grants made for Relief... | 28 | 15 | 8 | | | |
| " Grants made for Special Cases by Societies, &c. | 32 | 15 | 1 | | | |
| | | | | 61 | 10 | 9 |
| " Stamps and Postage ... | | | | 6 | 13 | 4 |
| " Stationery... ... | | | | 3 | 3 | 9 |
| " Books ... ... | | | | 3 | 4 | 9 |
| " Printing ... ... | | | | 11 | 5 | 3 |
| " Rent ... ... | | | | 41 | 0 | 0 |
| " Firing and Gas ... ... | | | | 5 | 7 | 6 |
| " Secretary's and Assistants' Salaries ... ... | | | | 99 | 0 | 0 |
| " Sundries ... ... | | | | 4 | 2 | 8 |
| " Balance in Bank ... ... | | | | 44 | 7 | 7 |
| | | | | £279 | 15 | 7 |

We have examined the above balance-sheet with the books and vouchers of the Society, and found it correct.

(Signed) W. T. Foster, E. Stopford Jones, } Auditors.

Of this total it will be seen that only £61 10s. 9d. was absolutely given for relief; £28 15s. 8d. fr:m the funds of the Association; £32 15s. 1d. given by other societies for special cases through the Friendly Workers. This is a point which, to my thinking, is of the first importance. People who have in any real way engaged in philanthropic work know that alms too often pauperise the recipients. This cannot be too strongly insisted on. Personally, therefore, I regard it as a matter of the very first importance that the North Kensington Friendly Workers should have shown themselves able to help, practically and substantially, seven hundred souls, over and above the large numbers of persons who have reaped benefits through the labour bureau, and in countless minor ways, with this exceedingly small amount of direct pecuniary relief; and the work has since September of last year (the date up to which the statistics given relate) very nearly repeated itself. That the sum spent in alms is so small compared with the total expenditure is, to my thinking, a matter to rejoice at, as being the proof that what was really given was time and care, by which means more good was done and less money spent. But there are people who like to do sums in this fashion: The money spent in relieving want was less than that thrown away (these good people like the expression "thrown away") on the salaries of the secretary and his assistants—less than half of the cost of these salaries and the rent of the office put together.

In answer to such complaints I would say, first (deprecatingly): The amount given as alms is as nothing to the money which, through the exertions of the secretary and his assistants, these poor people were enabled to earn. An estimate of that is unfortunately unattainable, or we should see a very considerable sum, which, moreover, has elevated those who obtained it, instead of pauperising them, and has given to those who paid it a satisfactory return in the shape of useful work done for them. The good done cannot be measured—not even materially, much less morally—by this paltry sum of £61 10s. 9d.

In the second place I would answer (this time defiantly) that the disproportion is not half as great as it should be. Mr. Mackenzie unfortunately accepted the post of secretary without salary. I say unfortunately, because an unpaid and devoted worker is always practically irreplaceable. Circumstances have now compelled Mr. Mackenzie to give up the work he has done so long and so well, and it is difficult to guess how he is to be replaced. Other men there may be as competent and as willing as he to give themselves to the work, to hold themselves day and night at the command of the poor and needy of North Kensington, but can it be assumed that any of these is at the same time in a position to give his services gratuitously? I do not quote the saying that the labourer is worthy of his hire; for there has never been a question of a remuneration that would be adequate for the energy, the care, the tact, the kindliness, which the position demands; but there has been a question of making it possible for a gentleman to give these things to the poor of the district without being himself reckoned among them. It has been suggested that the secretary should receive a salary of one hundred pounds a year, and there seems to be no prospect of raising that very modest sum.

(*To be continued.*)

Everyone will have noticed the terrible plague of flies we have had this year, and the "catch 'em alive oh!" men have driven a grand trade in consequence. We are inclined to regard flies as unpleasant rather than harmful, but reflection will point out that as carriers of contagion they are a real source of danger, and many cases of infection, the cause of which appears unaccountable, may be traceable to the agency of the domestic fly!

